# Sequential Changes in the Host Gut Microbiota During Infection With the Intestinal Parasitic Nematode *Strongyloides venezuelensis*

**DOI:** 10.3389/fcimb.2019.00217

**Published:** 2019-06-25

**Authors:** Tanzila Afrin, Kazunori Murase, Asuka Kounosu, Vicky L. Hunt, Mark Bligh, Yasunobu Maeda, Akina Hino, Haruhiko Maruyama, Isheng J. Tsai, Taisei Kikuchi

**Affiliations:** ^1^Division of Parasitology, Faculty of Medicine, University of Miyazaki, Miyazaki, Japan; ^2^Department of Environmental Parasitology, Tokyo Medical and Dental University, Tokyo, Japan; ^3^Biodiversity Research Center, Academia Sinica, Taipei, Taiwan

**Keywords:** host–parasite interaction, microbiome, *Strongyloides*, *Candidatus* Arthromitus, immune reaction

## Abstract

Soil-transmitted helminths (STHs) are medically important parasites that infect 1. 5 billion humans globally, causing a substantial disease burden. These parasites infect the gastrointestinal tract (GIT) of their host where they co-exist and interact with the host gut bacterial flora, leading to the coevolution of the parasites, microbiota, and host organisms. However, little is known about how these interactions change through time with the progression of infection. Strongyloidiasis is a human parasitic disease caused by the nematode *Strongyloides stercoralis* infecting 30–100 million people. In this study, we used a closely related rodent parasite *Strongyloides venezuelensis* and mice as a model of gastrointestinal parasite infection. We conducted a time-course experiment to examine changes in the fecal microbiota from the start of infection to parasite clearance. We found that bacterial taxa in the host intestinal microbiota changed significantly as the infection progressed, with an increase in the genera *Bacteroides* and *Candidatus* Arthromitus, and a decrease in *Prevotella* and *Rikenellaceae*. However, the microbiota recovered to the pre-infective state after parasite clearance from the host, suggesting that these perturbations are reversible. Microarray analysis revealed that this microbiota transition is likely to correspond with the host immune response. These findings give us an insight into the dynamics of parasite-microbiota interactions in the host gut during parasite infection.

## Introduction

Soil-transmitted helminths (STHs) are estimated to infect 1.5 billion people worldwide, 30–100 million of whom are parasitized by the nematodes *Strongyloides stercoralis* and *Strongyloides fuelleborni* (World-Health-Organization, 2017). The parasitic adult stages of *Strongyloides* spp. inhabit the gastrointestinal tract (GIT) of humans and other vertebrates (Viney and Lok, 2015) where they interact with the host gut microbiota, which can impact substantially on gut homeostasis. The microbial communities that colonize different regions of the human gut influence many aspects of health (Flint et al., 2012)—for example, they provide protection against enteropathogens, extract nutrients, and energy from our diets, and contribute to immune function (Lozupone et al., 2012). Therefore, an understanding of the gut microbiota and how it is influenced by parasitic nematode infection is important.

Two species of *Strongyloides* that parasitize rodents (*Strongyloides venezuelensis* and *Strongyloides ratti*) are well-established laboratory models for parasitic nematode infection although there are some limitations in utilizing those species as a model to study human *Strongyloides* infection mainly due to the absence of autoinfection cycle (Viney and Kikuchi, 2017). In *Strongyloides* spp., the life cycle alternates between free-living and parasitic generations (Hunt et al., 2016). After host skin penetration, the infective larvae of *S. venezuelensis* migrate to the GIT via the skin, muscles, and lungs (Takamure, 1995). Upon reaching the small intestine, adult parasitic females inhabit the mucosa of the duodenum and jejunum and produce eggs by parthenogenesis. The eggs then leave the host and enter the external environment via the host feces (Hino et al., 2014). During infection, the host mounts an immune response against the parasitic nematodes, leading to the expulsion of adult nematodes from the GIT at around 14 days post-infection (DPI) (Maruyama et al., 2002).

Recent studies have investigated how parasitic nematode infections affect the composition and diversity of the gut microbiota (Leung et al., 2018). However, changes in the microbiota composition with the progression of infection remain poorly understood. Time-series data can reveal interesting characteristics of the microbiome that will not be apparent when analyzing single time points, such as changes that occur at different stages of infection and the recovery of the microbiota after disturbance (Goodrich et al., 2014). Mice infected with *S. venezuelensis* represent a good experimental model for assessing sequential microbiota changes as infection progresses through to recovery because this parasite has a relatively short infection cycle (~20 days) compared with other intestinal parasites, whose cycles can last more than 3 months.

In this study, we conducted a time-course experiment to examine changes in the fecal microbiota of C57BL6 mice infected with *S. venezuelensis* from the start of infection until no parasite eggs could be detected in feces to gain a better understanding of the dynamics of parasite-microbiota interactions in the host gut.

## Materials and Methods

### Ethics Statement

All experiments were conducted in strict accordance with procedures that had been approved by the Animal Experiment Committee of the University of Miyazaki (Miyazaki, Japan) under approval no. 2009-506-6, as specified in the Fundamental Guidelines for Proper Conduct of Animal Experiment and Related Activities in Academic Research Institutions under the jurisdiction of the Ministry of Education Culture Sports Science and Technology, Japan, 2006.

### Biological Materials

Six-week-old male C57BL/6NJc1 mice were purchased from Kyudo (Saga, Japan) and held for 2 weeks in an animal housing unit in the parasitology laboratory of the University of Miyazaki before starting the experiments. The mice were maintained in individual cages under a 12 h light/dark cycle and at a constant temperature (25 ± 2°C) and were provided with dry pellets (CE-2; Feed-1 Company, Japan) and autoclaved drinking water. Each individual was transferred to a clean cage with fresh bedding material every morning and was monitored daily for signs of stress or disease throughout the experimental period. None of the mice developed restlessness, loss of appetite, diarrhea, or other intestinal-related issues during the experimental period.

*Strongyloides venezuelensis* single-female line HH1 was used in this study, which was originally isolated in Okinawa, Japan (Hino et al., 2014) and was maintained in the parasitology laboratory of the University of Miyazaki using male Wistar rats. Third-stage infective larvae (iL3) of *S. venezuelensis* were prepared by fecal culture using the filter paper method as described previously (Sato and Toma, 1990). Isolated iL3 were washed three times with phosphate-buffered saline (PBS) to remove any debris prior to inoculation.

### Experimental Outline

The experimental design is illustrated in Figure S1. Three mice were each infected subcutaneously with 1,000 *S. venezuelensis* iL3 while three additional naïve mice were left uninfected as a control. A fresh fecal sample was obtained from each infected mouse in the morning within 2 h of excretion in a clean cage every day from 0 (prior to infection) to 11 DPI, as well as at 14 and 17 DPI. In addition, fecal samples were collected from the control mice at 0, 5, and 11 DPI. Each fecal sample was frozen immediately after collection and stored at −80°C until use. The remainder of the feces were used to assess the worm burden of each individual [number of eggs per gram of feces (EPG)] by direct microscopic observation.

### DNA Extraction and Sample Preparation

DNA was extracted from the fecal samples using the cellulose magnetic bead-based extraction method (Afrin et al., 2018). In brief, approximately 0.2 g of feces from each sample was transferred into a 2.0 ml tube containing PowerBeads and 60 μl of solution C1 (MoBio). The sample was then homogenized using a Vortex-Genie2 Mixer (Scientific Industries) for 30 min at maximum speed followed by centrifugation at 10,000 × *g* for 2 min. The supernatant (~300 μl) was mixed with 30 μl of proteinase K and 300 μl of lysis buffer supplied with the Maxwell® RSC Blood DNA Kit (Promega) and was incubated at 56°C for 20 min, following which the solution was transferred to the Maxwell RSC Cartridge (Promega). Automatic DNA extraction was then performed with the Maxwell RSC instrument (Promega). The yield and purity of the extracted DNA were evaluated using NanoDrop (Thermo Fisher Scientific) and Qubit (Life Technologies) and the DNA integrity was assessed with electrophoresis using a 1% Tris-acetate-ethylenediaminetetraacetic acid (TAE) agarose gel.

### 16S rRNA Amplicon Sequencing

The extracted DNA was subjected to polymerase chain reaction (PCR) amplification targeting an approximately 300-bp fragment of the 16S rRNA variable region 4 using the universal bacterial primer set 515F/806R with barcode tags (Caporaso et al., 2012). PCR amplification was performed with the Ex Taq HS kit (Takara) in a thermal cycler (iCycler; BioRad) under the following conditions: 94°C for 3 min, followed by 25 cycles at 94°C for 30 s, 50°C for 60 s, and 72°C for 90 s, and a final extension step at 72°C for 10 min. PCR was performed independently for each duplicate DNA sample and the products were then mixed. The PCR products were purified using the MinElute 96 UF PCR Purification Kit (Qiagen) and quantified using NanoDrop 2000 (Thermo Fisher Scientific). All barcoded amplicons were pooled in equal concentrations and sequenced on the Illumina MiSeq platform using the MiSeq Reagent Nano Kit v2 (500 cycles) according to the manufacturer's recommended protocol (https://icom.illumina.com/) to produce 251-bp paired-end reads.

### Bioinformatics Analysis

The Illumina sequence data were processed using QIIME version 1.9.1 (Caporaso et al., 2010). The paired-end reads were first joined using the “fastq-join” method (join_paired_ends.py). QIIME quality filtering and library splitting were then carried out according to the Golay barcode sequences (split_libraries_fastq.py: -store_qual_scores -q 9 -max_barcode_errors 2 -sequence_max_n 1 -max_bad_run_length 2 -p 0.5 –r 3), following which chimeric sequences were detected with the UCHIME algorithm that is included in the free version of USEARCH61 and removed prior to further analysis. Cleaned reads were clustered and assigned to operational taxonomic units (OTUs) against the SILVA 132 database (Pruesse et al., 2007) with a 97% identity threshold using an open-reference OTU-picking protocol with “usearch” (pick_open_reference_otus.py: -s 0.5). OTUs were then further filtered from the *de novo* OTU table, removing taxa below a minimum fractional count of 0.01% (–min_count_fraction.00001).

Multiple rarefaction analysis was performed at a sequence depth of 50,000 using the QIIME script multiple_rarefaction.py to estimate each sequence depth was sufficient for diversity analysis. Alpha diversity was estimated by calculating the Chao1 index (Chao, 1984), Good's coverage and Shannon index using the QIIME script alpha_rarefaction.py. Beta diversity was assessed by calculating the Bray-Curtis dissimilarity index, which accounts for shared OTUs and abundance, using the QIIME script beta_diversity.py. Differences in community composition were visualized by performing principle coordinate analyses (PCoAs) in R using the Vegan package (Dixon, 2003).

The molecular functions of the bacterial communities were predicted using the Phylogenetic Investigation of Communities by Reconstruction of Unobserved States (PICRUSt) (Langille et al., 2013) based on 16S rRNA data with the KEGG database and Greengenes 13.5 reference taxonomy (Desantis et al., 2006). The 16S copy number was normalized using the normalize_by_copy_number.py script, molecular functions were predicted using the predict_metagenomes.py script and data were summarized into Kyoto encyclopedia of genes and genomes (KEGG) pathways and functional hierarchies using the categorize_by_function.py script, all of which are included in PICRUSt.

### Microarray Analysis

Mice were euthanized at 8 DPI and the anterior part of the duodenum (5–15 cm from the pylorus) was isolated and washed three times with PBS. RNA was then extracted from the duodenum using TriReagent (Molecular Research Center) according to the manufacturer's instruction. The RNA concentration and quality were evaluated using the Experion Automated Electrophoresis System (BioRad) and only RNA (100 ng) with an RNA Integrity Number (RIN) >7.5 was used for the microarray analysis. RNA labeling, microarray hybridization and scanning were performed using the Ambion WT Expression Kit (Thermo Fisher Scientific) and GeneChip Mouse Gene 1.0 ST Array (Affimetrix, Santa Clara, CA, USA) with the GeneChip Scanner 3000 (Affimetrix, Santa Clara, CA, USA), according to the manufacturer's instructions. Normalization and probe set summarization were performed on each sample separately using the Transcriptome Analysis Console Software v4.0 (Affimetrix). Genes were considered differentially expressed if they had a fold-change of at least 2 and a false discovery rate (FDR)-adjusted *p* < 0.05. Gene Ontology (GO) enrichment analysis for the differentially expressed genes was performed using the integrative web-based software GO Consortium with GO-slim and a significance level of <0.05 for the FDR-adjusted *p*-value. KEGG metabolic pathway analysis was performed using g-profiler (Raudvere et al., 2019).

### Statistical Analysis

Statistical tests were performed using R v 3.0.1 or STAMP software (Parks et al., 2014). Differences in the alpha diversity values between different time categories were tested using the Kruskal-Wallis test and Dunn's *post-hoc* test corrected for multiple comparisons with the Benjamini & Hochberg techniques. Differences in beta diversity were assessed by performing one-way analysis of similarity (ANOSIM) tests with Bray-Curtis distances (among different time categories and compared with control samples) using the compare_categories.py script implemented in QIIME.

Taxa in the microbiota that significantly changed through time were determined using the Kruskal-Wallis test and Dunn's *post-hoc* test, while the relationships between the abundance of *S. venezuelensis* (egg counts) and relative abundances of bacterial taxa were assessed using Spearman's rank correlations with the devtools package in R. Finally, differences in the predicted molecular functions of the bacterial communities were determined using Welch's *t*-test corrected with the Benjamini-Hochberg FDR. Differences were considered statistically significant when the *p* < 0.05.

## Results

### *Strongyloides venezuelensis* Infection Peaks at 8 DPI

**Figure 1:**
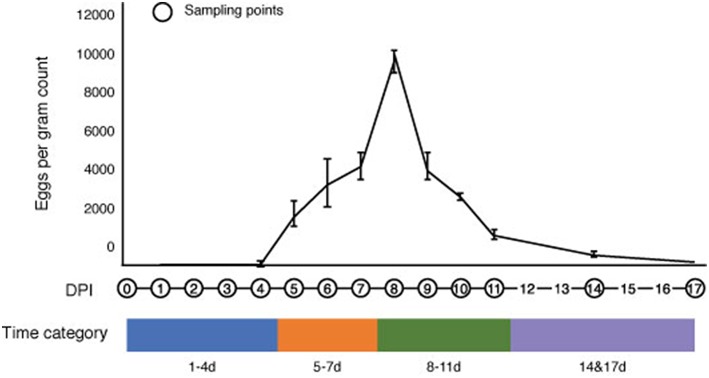
Changes in the number of *Strongyloides venezuelensis* eggs per gram of feces during the progression of infection. *S. venezuelensis* eggs were first detected at 5 days post-infection (DPI), with peak numbers being observed at 8 DPI. The infection period was divided into four categories: 1–4 DPI (blue), 5–7 DPI (orange), 8–11 DPI (green) and 14 and 17 DPI (purple).

We monitored the progression of *S. venezuelensis* infection in mice from the day of infection to the day of natural clearance (recovery) by calculating eggs in the feces (EPG) (Figure 1). No eggs were detected in the feces of infected mice at 1–4 DPI, which represents the period of larval migration through the host body to the small intestine. However, eggs were detected in the feces at 5 DPI, which corresponds with the expected time when nematodes would reach the intestine and start producing eggs (Takamure, 1995). The EPG peaked at 8 DPI, reaching a mean of 9,685 ± 571 eggs, and then decreased after this time until 17 DPI, when no parasite eggs were observed in the feces. Based on the status of infection, the time post-infection was separated into four categories for all further analyses: 1–4 DPI, representing the early stage of infection; 5–7 DPI, representing increasing EPG levels; 8–11 DPI, representing the peak and subsequent decrease in EPG; and 14 and 17 DPI, representing the post-infective recovery period (Figure 1).

### Gut Microbiota Diversity Is Reduced at the Peak of Infection

A total of 7,297,234 high-quality 16S rDNA reads were obtained from the fecal samples of the three infected mice collected every day from 0 to 11 DPI and at 14 and 17 DPI, with a mean of 155,260 reads per sample. The number of OTUs detected in each sample ranged from 1,271 to 1,718 with a mean of 1,585 (Table S1).

**Figure 2:**
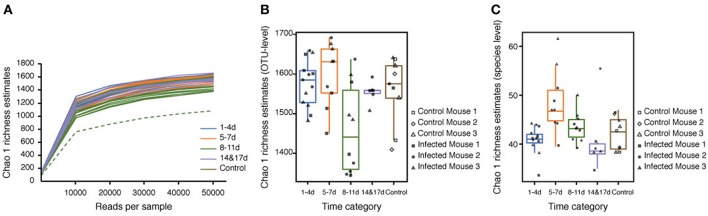
Alpha diversity of reads retrieved from the mouse fecal samples. **(A)** Rarefaction curves based on the Chao1 richness estimator calculated using the observed operational taxonomic units (OTUs). The total OTUs were generated using a 97% similarity threshold. **(B)** Box plot showing Chao1 values of bacteria in different time categories at the OTU level. **(C)** Box plot showing Chao1 values of bacteria in different time categories at the species level. Chao1 values were calculated using rarefied reads (50,000 reads per sample) from 0 to 17 days post-infection (DPI), as well as for the naïve control mice.

Rarefaction curves generated for the Chao1 richness estimator nearly reached asymptotes at read depths of 50,000 for all samples, indicating that the sampling depth provided sufficient coverage for a comprehensive analysis of the bacterial composition of each sample (Figure 2A). Furthermore, Good's coverage was >0.97 for all of the samples, implying that the depth of coverage was sufficient (Table S1). One sample (Infected Mouse 1 at 11 DPI) was treated as an anomaly and excluded from downstream analysis due to its low Chao1 value compared with the other replicates. The rarefaction curves indicted that the species richness was lower at 8–11 DPI than for the other time categories (Figure 2A) and a comparison of the Chao1 values by time categories showed that the species richness was significantly lower at 8–11 DPI compared with the control (Dunn's *post-hoc* test: *Z* = −2.44, *p* < 0.04), 1–4 DPI (*Z* = 3.08, *p* < 0.02), and 5–7 DPI (*Z* = 2.75, *p* < 0.02) samples (Figure 2B, Table S2). By contrast, the median Chao1 value was higher during the recovery period (14 and 17 DPI) than at 8–11 DPI, though this difference was not statistically significant, and the range of the Chao1 values was also reduced during the recovery period. There was, however, no significance difference in the Chao1 index based on species-level OTUs (i.e., assigned species composed of single or multiple OTUs) between samples in the 8–11 DPI category and those in the other time categories (Dunn's *post-hoc* test: *p* > 0.21) (Figure 2C) and the Shannon index was found to be stable across all samples, with no statistically significant difference between the time categories (Kruskal-Wallis H test: χ^2^ = 1.8592, df = 4, *p* = 0.76).

A diverse range of bacterial phyla were identified in the fecal samples, including Bacteroidetes, Firmicutes, Tenericutes, Proteobacteria, Cyanobacteria, and Actinobacteria (Figure S2). In the control samples, Bacteroidetes were most prevalent, followed by Firmicutes, Tenericutes, and Proteobacteria, together representing >97% of the total microbiota, which is consistent with previous observations in healthy C57BL/6 mouse GITs (Lozupone et al., 2012) and fecal samples (Holm et al., 2015; Houlden et al., 2015). The relative proportions of these phyla were mostly maintained in the infected samples, with no significant changes being seen at the phylum level between the different time categories (Kruskal-Wallis H test: χ^2^ = 4, df = 4, *p* = 0.406) (Figure S2).

A clear shift in microbial proportions was seen at the family level, however. The relative abundance of families belonging to the phylum Bacteroidetes showed significant differences between samples in the 8–11 DPI category and control samples, with significant increases in *Bacteroidaceae* (Dunn's test: *Z* = 3.3, *p* = 0.007) and significant decreases in *Rikenellaceae* (*Z* = −3.3, *p* = 0.004), *Paraprevotellaceae* (*Z* = −4.1, *p* = 0.0003), *Prevotellaceae* (*Z* = −4.0, *p* = 0.0004), and the *S24-7* group of uncultured Bacteroidetes (*Z* = −3.9, *p* = 0.0003). In addition, the relative abundance of the family *Clostridiaceae* (phylum Firmicutes) was also significantly higher in the 8–11 DPI category compared with the control (*Z* = 3.2, *p* = 0.003).

**Figure 3:**
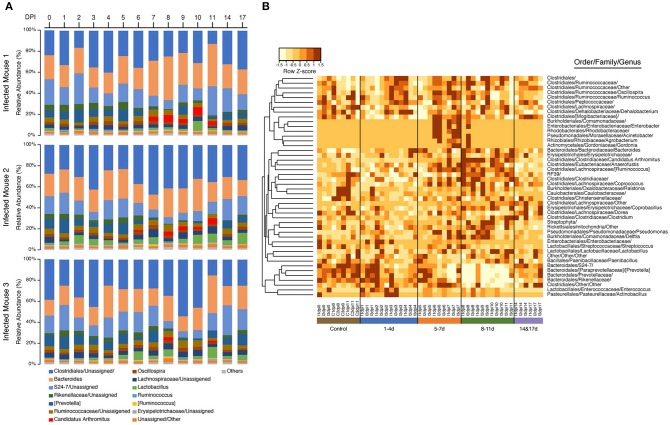
Impact of *Strongyloides venezuelensis* infection on the gut bacterial community composition. **(A)** Relative abundance of the fecal microbiota at the genus level in infected mice. The taxonomic composition and relative abundance of bacterial genera in the fecal samples with the progression of *S. venezuelensis* infection are shown. Taxa with relative abundances of <1% are included in “Others.” **(B)** Heatmap of the operational taxonomic unit (OTU) abundance in individual mice demonstrating large-scale community shifts at 5–7 and 8–11 days post-infection (DPI). Each column represents an individual sample from the control or infected mice during infection, while each row represents a genus-level OTU Values were normalized by row.

**Figure 4:**
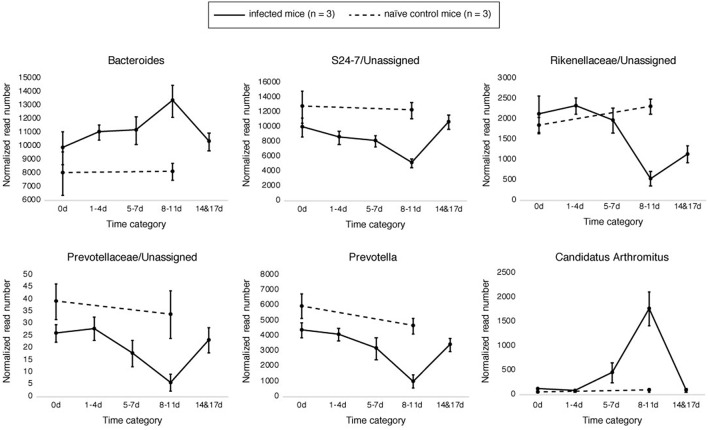
Relative abundances of the operational taxonomic units (OTUs) that significantly changed during the infection period. Six genera were found to have significantly changed in abundance at 8–11 days post-infection (DPI) relative to the control: *Bacteroides* (Bacteroidetes), *Prevotella* (Bacteroidetes), *S24-7* (Bacteroidetes), an unclassified genus derived from *Rikenellaceae* (Bacteroidetes), *Prevotellaceae* (Bacteroidetes), and *Candidatus* Arthromitus (Firmicutes). Solid and break lines represent the naïve control mice (*n* = 3) and the infected mice (*n* = 3), respectively.

**Figure 5:**
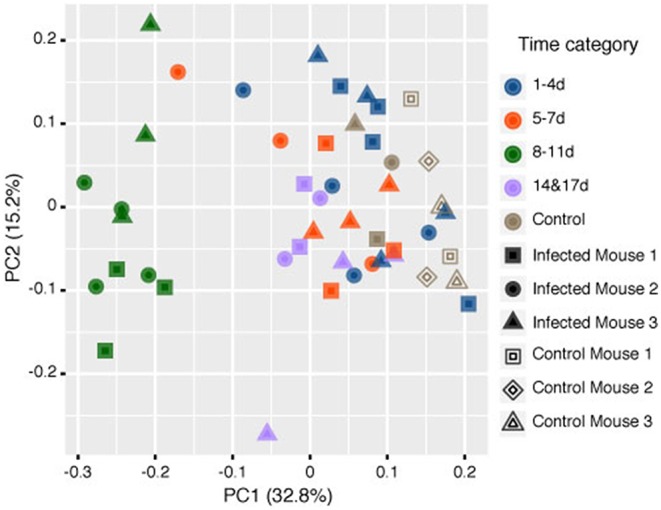
Principle coordinate analysis (PCoA) of the rarefied operational taxonomic units (OTUs) comparing the microbiome contents of mice infected with *Strongyloides venezuelensis* from 0 to 17 days post-infection (DPI) and naïve control mice based on the Bray-Curtis dissimilarity distances Analysis of similarities (ANOSIM) tests showed that the samples at 8–11 DPI were significantly different from the samples in the other time categories and the control groups (*p* = 0.001).

Samples in the 8–11 DPI category also differed from those in the other time categories at the genus level, with specific groups of taxa increasing or decreasing in abundance during the course of infection (Figures 3A,B). Significant increases in the relative abundances of the genera *Bacteroides* (Dunn's test: *Z* = 3.3, *p* = 0.007) and *Candidatus* Arthromitus (*Z* = 3.70, *p* = 1.0e-03) were observed in the 8–11 DPI category compared with the control samples, whereas significant decreases were observed in the genera *Prevotella* (*Z* = −4.1, *p* = 0.0003), an unclassified genus derived from *Rikenellaceae* (*Z* = −3.3, *p* = 0.004), *S24-7* (*Z* = −3.9, *p* = 0.0007), and *Prevotellaceae* (*Z* = −4.1, *p* = 0.0004) (Figure 4). Consistent with these observations, PCoA plots of the Bray-Curtis distances showed a clear separation between the 8–11 DPI category and all other time categories (ANOSIM: *p* = 0.001) (Figure 5). By contrast, no clear separations were seen between the 1–4, 5–7, and 14 and 17 DPI categories and their respective controls. However, several taxa belonging to the phylum Proteobacteria significantly increased in abundance at 5–7 DPI, including the genera *Agrobacterium* (Dunn's test: *Z* = 3.8, *p* = 0.0006), *Enterobacter* (*Z* = 3.1, *p* = 0.02), *Pseudomonas* (*Z* = 2.7, *p* = 0.03), unclassified genera of the family *Rhodobacteraceae* (*Z* = 3.5, *p* = 0.002), and *Comamonadaceae* (*Z* = 3.1, *p* = 0.0004), although these fraction amounts were small (Figure S3).

### Bacterial Relative Abundance Is Weakly Correlated With Egg Counts

To investigate the relationship between the presence of parasite eggs and changes in the gut microbiota, we examined the correlations between the number of *S. venezuelensis* eggs and the relative abundance of each bacterial taxon in the mouse feces. Overall, we found that these correlations were not very strong. Only three of the 46 bacterial taxa identified showed strong correlations with egg counts (rho > 0.5 or < -0.5) across all mouse replicates (Table S3): positive correlations were observed for *Candidatus* Arthromitus and *Agrobacterium*, while a negative correlation was found for an unclassified genus of the family *Prevotellaceae*. In addition, we found that the number of *S. venezuelensis* eggs was positively correlated with the abundances of six genera and negatively correlated with the abundance of one genus in two of the three replicates.

### Metabolic Capacity of the Fecal Microbiota Changes at the Peak of Infection

To correlate the fecal microbiota composition data and inferred changes in bacterial metabolism, in response to the parasitic helminth infection, we conducted a predictive metagenomics analysis using PICRUSt. Functional estimation of the metagenomic profile suggested that there were significant differences in the metagenomic functional content of the fecal microbiota between the 8–11 DPI category and the control. At KEGG level II, signal transduction and carbohydrate metabolic pathways were enriched in the 8–11 DPI category relative to the control, whereas genes involved in amino acid metabolism, translation, nucleotide metabolism, and neurodegenerative disease pathways were significantly decreased. At KEGG level III, pathways associated with pentose-glucuronate interconversions and the pentose phosphate pathway (PPP) were enriched in the 8–11 DPI category compared with the control, while pathways associated with amino acid metabolism as well as proteolysis were significantly underrepresented (Figure S4).

### Genes Involved in Host Immune Response and Muscle Contraction Were Upregulated During *S. venezuelensis* Infection

**Figure 6:**
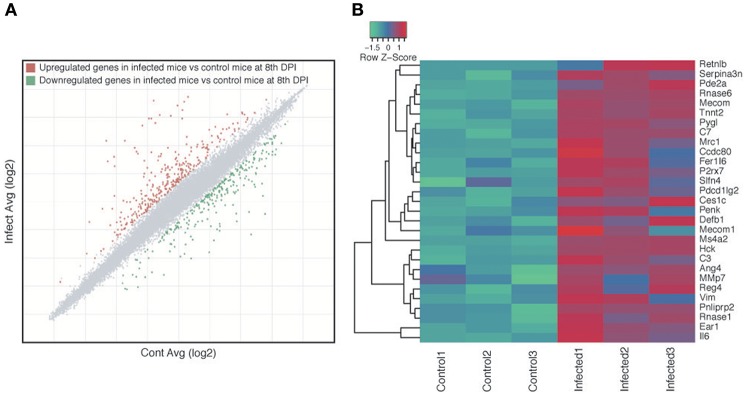
Host gene expression changes as a result of *Strongyloides venezuelensis* infection. **(A)** Scatter plot highlighting the genes that were differentially expressed between infected and naïve control mice at 8 days post-infection (DPI). Each dot represents a gene, with gray, red, and green dots indicating non-differentially expressed, upregulated and downregulated genes, respectively, in the infected mice relative to the control mice. **(B)** Heat map of all 29 genes with the Gene Ontology (GO) term “responded to the bacteria” (GO:0009617).

We investigated if the host immune response may be one of the factors that is directly or indirectly involved in altering the microbiota compositions during parasite infection. Microarray analyses of the host intestines showed that 593 genes were differentially expressed in the small intestines of infected mice relative to the control mice, 329 of which were upregulated and 264 of which were downregulated (Figure 6A, Table S4). GO enrichment analysis revealed that 228 biological processes, 42 cellular components and 13 molecular functions were modulated during parasite infection (Table S5). The enriched biological process GO terms for upregulated genes in the infected mice included immune response, immune system process, response to external stimulus, and muscle contraction (Table S5), with genes that are assigned to GO terms involved in the response to bacteria being particularly upregulated in the infected samples (Figure 6B). Enriched GO terms for downregulated genes included various types of metabolic process such as lipid metabolic process, secondary metabolic process, prostaglandin metabolic process, and gluconeogenesis (Table S6). Consistently, KEGG biological pathway analysis showed that immunity-related pathways including chemokine, Fc epsilon RI and B cell receptor signaling pathway were enriched with upregulated genes (Table S7). Several metabolic pathways such as retinol metabolism, drug metabolism, steroid hormone biosynthesis, and amino-acid metabolisms were enriched with downregulated genes (Table S8).

These results suggest that *S. venezuelensis* infection altered the physiology and gene expression within the host intestine, particularly in relation to the immune response, muscle contraction, bacterial response as well as a variety of metabolisms, which may affect the intestinal microbiota. In addition, the host may react to these changes in the microbiota, which may reduce or enhance them.

## Discussion

In this study, we performed a time-series analysis of the fecal microbiota of mice infected with the parasitic nematode *S. venezuelensis* to improve our understanding of how the host microbiota changes during infection. A healthy gut microbial ecosystem is generally thought to be characterized by a high bacterial richness and diversity, as this is presumed to reflect ecosystem stability and resilience (Cho and Blaser, 2012; Lozupone et al., 2012). Several inflammatory conditions (Papa et al., 2012; Degruttola et al., 2016; Zechner, 2017) and parasite infections (Holm et al., 2015; Cattadori et al., 2016; Jenkins et al., 2018b) have been shown to be associated with a reduced species richness in the murine model. Similarly, in the present study, we found that the bacterial species richness estimate (Chao1) declined in the middle phase of infection (8–11 DPI), reflecting temporary microbial dysbiosis in the host, possibly due to intestinal immune responses. This change appears to have been caused by rare OTUs in certain taxa rather than major bacterial taxa, with no significant differences being observed at the species level. By contrast, although Jenkins et al. (2018a) did not observe any significant differences in species richness during *Strongyloides* infection in humans, they did observe a trend toward increased richness in helminth-positive samples. Similar differences have also been observed for other helminth species. For example, while *Trichuris* infection in humans was found to be associated with an increased diversity (Cooper et al., 2013), the infection resulted in a significant reduction in microbiota diversity in a well-controlled laboratory experiment with mice (Holm et al., 2015; Houlden et al., 2015). Furthermore, *Schistosoma* infection does not significantly affect the species richness in humans (Schneeberger et al., 2018) but reduces it in a murine model of infection (Jenkins et al., 2018b). These results indicate that microbiota diversity is affected not only by the parasite infection but also by other environmental and host genetic factors, as well as the type (acute or chronic) and dose of the infection. In addition, the stage of infection may also influence the microbiota, as seen in our time-series experiments.

Changes in the intestinal microbiota of infected mice are likely due to interactions between three players: the host, the infecting parasites and the host gut microbes. Our results suggested that while the presence of parasite eggs in the feces could be one of the factors that alter the fecal microbiota, other factors are likely to be more important, such as the host immune response or parasite-secreted products. *S. venezuelensis* infection induces T-helper type 1 (Th1) and Th2 immune responses in the host duodenum and colon (Rodrigues et al., 2018), with Th2 responses in particular causing changes to the intestinal physiology, including increased mucosal permeability, enhanced smooth muscle contractility, and an altered microbiota (Zhao et al., 2003; Artis and Grencis, 2008; Farid et al., 2008; Su et al., 2011). In the present study, we found that Th2-related genes were upregulated in the middle phase of infection. A parasite-induced immune response can trigger disequilibrium of the microbiota by inducing compositional changes in the mucus layer, i.e., the region of the intestine that is inhabited by *S. venezuelensis*, or by damaging junctional proteins in the epithelial layer (Maruyama et al., 2002). Consequences of the “leaky gut” could be secondary infection, sepsis and bacterial translocations, which may induce a systemic inflammation in the host (Farid et al., 2008).

In this study, microarray analysis of infected host tissue at 8 DPI, the time point when infection peaked and the greatest change of microbiota was observed, showed an increase in immune-related gene expression. This confirms that changes in the host are also occurring at 8 DPI. Further studies investigating gene expression at different time points would reveal how the immune response changes as infection progresses and be useful to further understand host-parasite-microbe interactions. Based on previous studies it is likely that the immune response changes over the course of infection. For example, histo-immunological studies reported a dynamic change of immune response during *Strongyloides* infection. For example, mast cells, leukocytes and eosinophils begin to increase immediately after infection (~2 DPI), peaked at 7–8 DPI and decreased in recovery phase (~12 DPI) (Silveira et al., 2002; Sasaki et al., 2005) whereas IgG and IgE levels peaked at 14 DPI (Matsumoto et al., 2013).

The direct interaction between parasites and microbes in the GIT could also cause parasite-induced changes in the microbiota. Parasitic helminths secrete products that have bactericidal or bacteriostatic activities, which may create a favorable environment for their survival (Cotton et al., 2012; Cooper and Eleftherianos, 2016). Antimicrobial peptides have been identified in various nematodes, such as *Caenorhabditis elegans* (Dierking et al., 2016) and *Ascaris suum* (Kato and Komatsu, 1996; Midha et al., 2018), as well as a number of trematodes, including *Fasciola* spp. and *Schistosoma* spp. (Cotton et al., 2012). In addition, neuropeptide-like proteins with conserved YGGYG motifs (Gravato-Nobre and Hodgkin, 2005) are secreted by a wide variety of nematodes, including *S. ratti*, which also infects murines, and have been shown to have antibacterial activity (Gravato-Nobre and Hodgkin, 2005; Mcveigh et al., 2008). We identified neuropeptide-like protein (*nlp*) genes with these motifs in the *S. venezuelensis* genome and also recently revealed that *S. venezuelensis* secretes a mixture of a wide variety of proteins in the host small intestine, including peptidases and histones, which likely have antimicrobial activities (Maeda et al., 2019). Therefore, these proteins may directly interact with the gut microbiota, leading to dysbiosis in the host gut.

*Candidatus* Arthromitus spp., which are also known as segmented filamentous bacteria (SFB), are well-known for their immunomodulatory effects (Umesaki et al., 1995; Talham et al., 1999) and are specific inducers of Th17 cell differentiation (Ivanov et al., 2009). Members of the genus *Bacteroides* also influence the host immune system and control other (competing) pathogens, as well as having the highest antibiotic resistance rates among all anaerobic pathogens (Wexler, 2007). It has recently been reported that the increased abundance of specific gut microbes, such as Bacteroides and SFB, during *Heligmosomoides polygyrus* infection is Th2-response dependent (Rausch et al., 2013; Su et al., 2018). Here, we observed an increase in *Candidatus* Arthromitus and *Bacteroides* in the middle of the *S. venezuelensis* infection period, which we assume was also caused by the Th2 response. Interestingly, no such increase was observed for the genus *Lactobacillus* in the present study, which contrasts with other recent studies on murine–helminth infections (Reynolds et al., 2014; Holm et al., 2015;Minamoto et al., 2015).

Functional estimation of the bacterial metagenomic profile suggested that there was an enrichment of microbiomical capacity of the pentose phosphate pathway in the middle of the *S. venezuelensis* infection. This pathway is an alternative to glycolysis and a critical pathway for the cellular redox balance and protection from oxidative stress (Riganti et al., 2012). Upregulation of this pathway in GIT microbiota may represent a response to an oxidative stress in the host environment during *S. venezuelensis* infection. An association between the alteration of GI microbial communities and the presence of oxidative stress in the GIT has also been reported in human inflammatory bowel diseases (IBD) patients (Minamoto et al., 2015). The enriched biosynthesis of siderophore group non-ribosomal peptides may also be due to the depletion of local iron availability, which induces the upregulation of siderophore systems by resident and pathogenic bacteria and enables the efficient scavenging of iron (Ellermann and Arthur, 2017). In addition, changes of both microbiomical capacity and host gene expression were observed in amino-acid metabolic pathways during *S. venezuelensis* infection. A decreased lysine biosynthesis and tryptophan metabolism have been reported during intestinal inflammation (Corfield et al., 2000; Tsune et al., 2003; Morgan et al., 2012). It has also been reported that *Plasmodium* parasite depletes the host arginine pool in order to modulate the activity of the host enzyme which suppress the immune system (Tachado et al., 1996; Olszewski et al., 2009). Therefore, the changes observed in *S. venezuelensis* infection may also be the result of inflammation or particular types of immune response. It remains to be investigated through comprehensive metabolome and transcriptome studies why these metabolic changes occur during the infection, but these results clearly suggest *S. venezuelensis* infection induces host immune responses and disrupts gut homeostasis. The host-parasites-microbe interactions in the infected GIT are complex; some of which lead to dysbiosis and others may keep the homeostatic balance.

In conclusion, this study provides a comprehensive view of changes in the gut microbiota during the course of a parasitic nematode infection. In contrast to previous studies that have used limited time points for sampling, this time-series experiment analyzed the gut microbiota on a daily basis to elucidate the contribution of parasite-associated modifications and host immune responses to microbiota dynamics in the host intestine. Mice are not a natural host of *S. venezuelensis* and the results might not be as easily compared with the infection of rats (the known natural host) (Viney and Kikuchi, 2017). This study, however, doubtfully deepen our understanding of the complex interactions between the host, parasite and microbiota as mice have long been used as a laboratory host of this parasite and there have been extensive immunological analyses of its infections in mice (Wilkes et al., 2007). Our results suggests that, although the microbiota dysbiosis that is caused by the parasite infection is reversible, chronic infections that lead to continuous microbiota dysbiosis may increase the host's susceptibility to future diseases, such as colitis and IBD, and can also have more adverse effects on immunocompromised patients.

## Data Availability

The sequencing data have been deposited in the DNA Data Bank of Japan (DDBJ) Sequence Read Archive under the BioProject SAMD00148911. The microarray CEL files and normalized data have been deposited into the DDBJ Genomic Expression Archive (GEA) repository under accession number E-GEAD-288.

## Ethics Statement

All experiments were conducted in strict accordance with procedures that had been approved by the Animal Experiment Committee of the University of Miyazaki (Miyazaki, Japan) under approval no. 2009-506-6, as specified in the Fundamental Guidelines for Proper Conduct of Animal Experiment and Related Activities in Academic Research Institutions under the jurisdiction of the Ministry of Education Culture Sports Science and Technology, Japan, 2006.

## Author Contributions

IT and TK conceived and designed the experiments. TA, AK, and AH performed the experiments. TA, KM, VH, IT, and TK analyzed the data. TA, VH, MB, and TK wrote the paper. YM, KM, IT, and HM provided critical comments and revised the paper. All authors read and approved the final manuscript.

### Conflict of Interest Statement

The authors declare that the research was conducted in the absence of any commercial or financial relationships that could be construed as a potential conflict of interest.
