# Supplementary material for: Sequential Changes in the Host Gut Microbiota During Infection With the Intestinal Parasitic Nematode Strongyloides venezuelensis
Source: Front Cell Infect Microbiol. 2019 Jun 25;9:217. doi: 10.3389/fcimb.2019.00217 (PMC6604662; doi:10.3389/fcimb.2019.00217)

FIGURE S1 | Experimental outline for monitoring changes in the faecal microbiota of mice infected with *Strongyloides venezuelensis* (*n* = 3) and naïve control mice (*n* = 3).

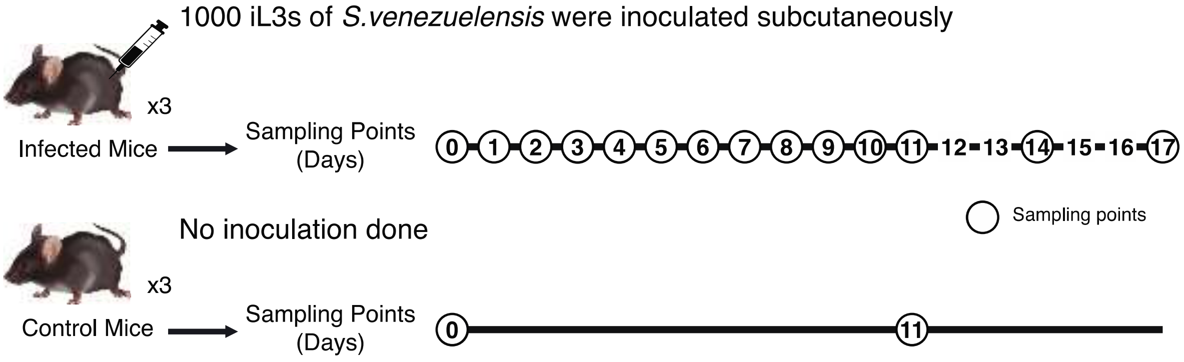

FIGURE S2 | Relative abundance of the faecal microbiota at the phylum level in mice infected with *Strongyloides venezuelensis*. The taxonomic composition and relative abundance are shown at the phylum level, with each bar representing an individual mouse's microbial community in the faeces sampled from 0 to 11 days post-infection (DPI) and at 14 and 17 DPI.

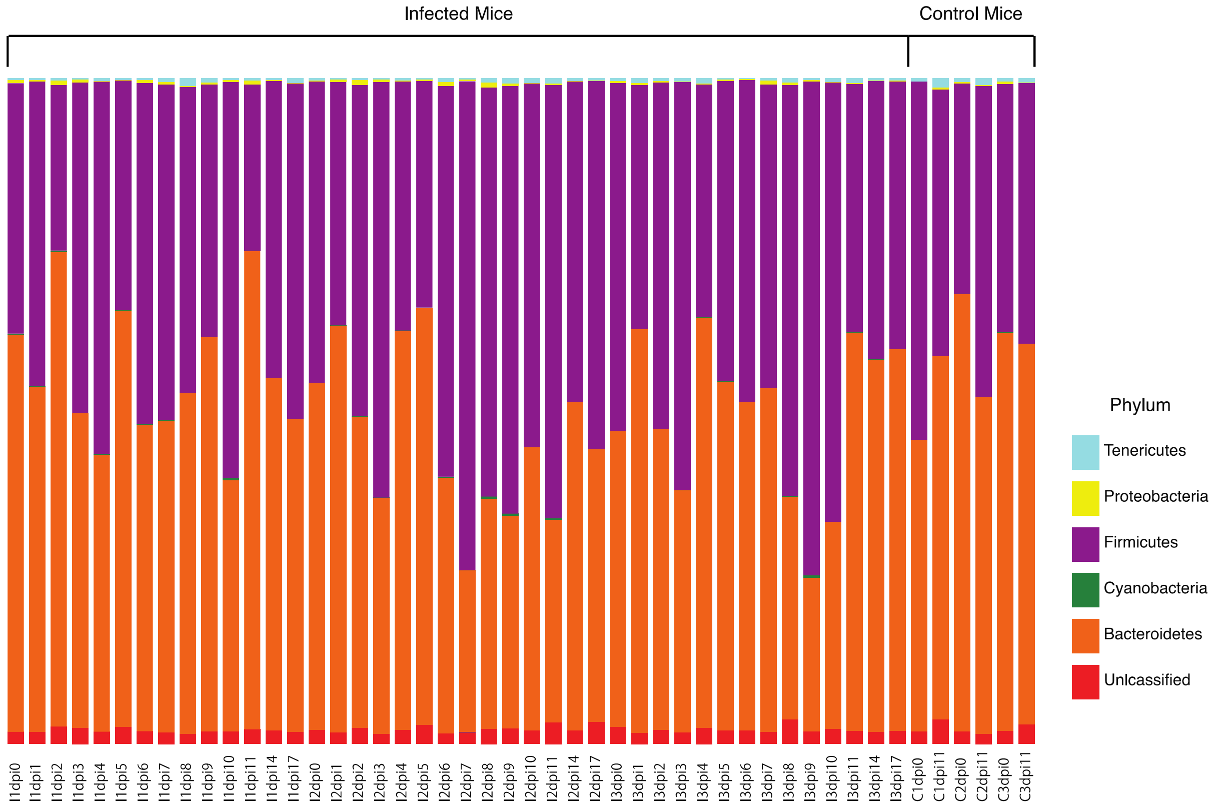

Figure S3 | Relative abundances of operational taxonomic units (OTUs) that significantly changed during the disease progression. The abundances of five genera were significantly different at 5–7 days post-infection (DPI) compared with the control: *Agrobacterium* (Proteobacteria), *Pseudomonous* (Proteobacteria), *Enterobacter* (Proteobacteria), an unclassified genus derived from *Comamonadaceae* (Proteobacteria) and *Rhodobacteraceae* (Proteobacteria). Solid and break lines represent the naïve control mice (n = 3) and the infected mice (n = 3), respectively.

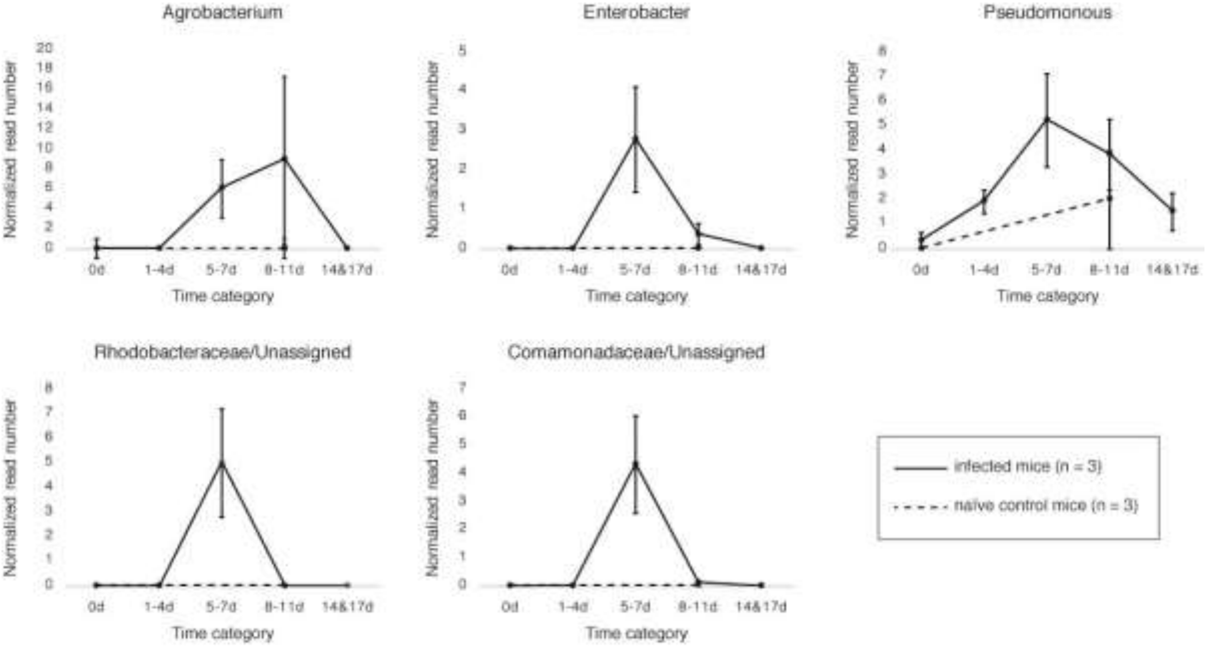

FIGURE S4 | Predicted functional pathway analysis of the faecal microbiota in mice infected with *Strongyloides venezuelensis*. The predicted functional pathways for the faecal microbiota are compared between mice at 8–11 days post-infection (DPI) and naïve control mice. The *q*-values are based on Welch's *t*-test and corrected with the Benjamini-Hochberg false discovery rate (FDR).

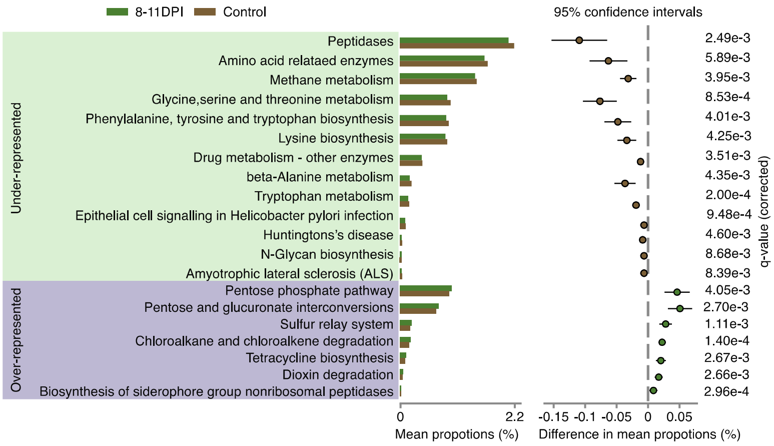

Supplement: Supplementary file 1 [file Data_Sheet_1.PDF]
